# Implementing peer support into practice in mental health services: a qualitative comparative case study

**DOI:** 10.1186/s12913-024-11447-5

**Published:** 2024-09-11

**Authors:** Steve Gillard, Rhiannon Foster, Sarah White, Rahul Bhattacharya, Paul Binfield, Rachel Eborall, Sarah L Gibson, Daniella Harnett, Alan Simpson, Mike Lucock, Jacqueline Marks, Julie Repper, Miles Rinaldi, Anthony Salla, Jessica Worner

**Affiliations:** 1 City, University of London, London, UK; 2 St George’s, University of London, London, UK; 3 East London NHS Foundation Trust, London, UK; 4 South London & Maudsley NHS Foundation Trust, London, UK; 5 Kingston University, Kingston, UK; 6 King’s College London, London, UK; 7 University of Huddersfield, Huddersfield, UK; 8 Kingston Hospital, Kingston, UK; 9 Implementing Recovery through Organisational Change, Nottingham, UK; 10 South West London & St George’s Mental Health NHS Trust, London, UK; 11 Nordland Hospital Trust, Bodø, Norway; 12 Independent Researcher, London, UK

**Keywords:** Peer support, Mental health services, Lived experience, Role adoption, Implementation, Comparative case study methods.

## Abstract

**Background:**

Peer workers are people with personal experience of mental distress, employed within mental health services to support others with similar experiences. Research has identified a range of factors that might facilitate or hinder the introduction of new peer worker roles into mental health services. While there is mixed evidence for the effectiveness of peer worker delivered interventions, there are no studies exploring how implementation might be associated with effect.

**Methods:**

This was a qualitative comparative case study using data from interviews with 20 peer workers and their five supervisors. Peer workers delivered peer support for discharge from inpatient to community mental health care as part of a randomised controlled trial. In the trial, level of participant engagement with peer support was associated with better outcome (hospital readmission). Study sites with higher levels of engagement also had higher scores on a measure of fidelity to peer support principles. We compared data from sites with contrasting levels of engagement and fidelity using an analytical framework derived from implementation theory.

**Results:**

In high engagement-high fidelity sites, there was regular work with clinical teams preparing for working alongside peer workers, and a positive relationship between staff on inpatient wards and peer workers. The supervisor role was well resourced, and delivery of peer support was highly consistent with the intervention manual. In low engagement-low fidelity sites peer workers were employed in not-for-profit organisations to support people using public mental health services and in rural areas. Supervisors faced constrained resources and experienced barriers to joint working between organisations. In these sites, peer workers could experience challenging relationships with ward staff. Issues of geography and capacity limited opportunities for supervision and team-building, impacting consistency of delivery.

**Conclusions:**

This study provides clear indication that implementation can impact delivery of peer support, with implications for engagement and, potentially, outcomes of peer worker interventions. Resourcing issues can have knock-on effects on consistency of delivery, alongside challenges of access, authority and relationship with clinical teams, especially where peer workers were employed in not-for-profit organisations. Attention needs to be paid to the impact of geography on implementation.

**Trial registration:**

ISRCTN registry number ISRCTN10043328, registered 28 November 2016.

**Supplementary Information:**

The online version contains supplementary material available at 10.1186/s12913-024-11447-5.

## Background

### Peer support in mental health services

People with personal experience of mental distress, often referred to as peer workers, are increasingly employed within mental health services internationally to support others with similar experiences. An extensive literature explores a range of implementation issues that might dilute the distinctive qualities of peer support when introduced into public mental health services [1, 2]. These include adequate provision of role specific training for PWs, [3, 4] support and supervision for PWs, [5] clarity of expectation around the way in which PWs bring experience-based knowledge to mental healthcare, [2.^6^] and preparation of clinical teams to work alongside PWs [7]. It has been argued that ‘over-professionalisation’ or ‘institutionalisation’ of the PW role constrains the distinctive contribution of peer support [8–11].

Trials of peer support in mental health services continue to demonstrate inconsistent results, with some studies indicating that peer support might be superior to care-as-usual or a comparator intervention, [12, 13] while others indicate no difference in effect [14, 15]. Some of this variation might be explained by heterogeneity of interventions, population or outcome, but it is also possible that the quality of implementation of peer support into mental healthcare settings is associated with the effect of peer support interventions [16, 17].

It has been noted that peer support is often poorly described in the trial literature, [17, 18] with a lack of research assessing association between implementation and outcome. A recent review of one-to-one peer support in mental health services categorised peer support as being well implemented where at least two of the following criteria were reported: dedicated peer support training; clear description of the underlying processes of peer support; well-defined support structures for PWs (e.g. supervision) [19]. However, only a small number of studies reported sufficient data to conduct an analysis and results were unclear. There is a need for research that explicitly considers the possible relationship between quality of implementation and the outcomes of peer support.

### Implementation theory

Implementation science offers a range of frameworks for understanding the facilitators and barriers to successful implementation of healthcare innovation into practice [20]. There is a clear recognition that the effects of any intervention will always depend on successful implementation [21]. The well-established Promoting Action on Research Implementation in Health Services (PARIHS) framework conceptualises successful implementation of research-based innovation into healthcare in terms of the nature of the evidence on which the innovation is based, the context or environment into which the innovation is placed, and the method by which implementation is facilitated [22]. In recent years, the co-design [23] or coproduction [24] of new interventions in mental health has gained prominence, with people who use mental health services bringing experience-based knowledge to the process, alongside the professional and practice-based knowledge brought by healthcare professionals. Given that this experiential knowledge is core to peer support, and that a number of members of the research term brought their own experiences of mental distress and/ or of using mental health services to the design and conduct of the research, we adapted the PARIHS framework for the purposes of this study. An earlier scoping review of implementation literature and an empirical case study, [25] undertaken by members of the team (SG and RF), identified five domains where experiential knowledge might impact research implementation, and we mapped these domains directly onto the framework (Table 1).

**Table 1:** PARIHS framework adapted to incorporate the role of experiential knowledge in successful implementation

	Low	High
**Evidence**		
Research	Anecdotal evidence Descriptive information	Randomised controlled trials Systematic reviews Evidence based guidelines
Clinical experience	Expert opinion divided Several camps	High levels of consensus Consistency of views
Patient experience	Patients not involved	Partnerships
*Relational models of knowledge production*	*People using health services on the receiving end of research knowledge* *Traditional clinical academic research teams*	*People using health services (co)producers of research knowledge* *People using health services leading or core members of research teams*
*Experiential knowledge*	*Formal*,* codified research knowledge dominates* *Experiences re-constructed by clinical academic discourse* *Distant from service user audience*	*Experiential*,* tacit knowledge recognised as a valid source of evidence* *Counterbalances or critiques clinical knowledge* *Relevant to service user audience*
**Context**		
Culture	Task driven Low regard for individuals Low morale Little of no continuing education	Learning organisation Patient centred Valuing people Continuing education
*Implementation context*	*Organisation not receptive to challenge and change*	*Organisation receptive to change that challenges culture* *Partner organisations bring cultural challenge*
Leadership	Diffuse roles Lack of team roles Poor organisation or management of services Poor leadership	Clear roles Effective team work Effective organisational structure Clear leadership
Measurement	Absence of: Audit & feedback Peer review External audit Performance review	Internal measurement used routinely Audit or feedback used routinely Peer review *including from a experiential perspective* External measures
**Facilitation**		
Characteristics	Low levels of respect, empathy, authenticity & credibility	High levels of respect, empathy, authenticity & credibility
Role	Lack of clarity around: Access Authority Position in organisation Change agenda	Access Authority Change agenda successfully negotiated
Style	Inflexible Sporadic Infrequent Inappropriate	Range & flexibility of style Consistent & appropriate presence & support
*Collaborative practice*	*Production and transfer of research knowledge preserve of academic team*	*People providing and using services involved in production and transfer of research knowledge*
*Knowledge facilitation*	*Lack of engagement across boundaries between stakeholders* *Research outputs distant from practice and service user experience*	*Individuals with lived experience/ peer organisations act as knowledge brokers between stakeholders* *Coproduction of research outputs that are relevant and practical*

Adapted from Kitson, A., Harvey, G., & McCormack, B. (1998). Enabling the implementation of evidence based practice: a conceptual framework. BMJ Quality & Safety, 7(3), p.151. Text in italics is in addition to the original framework and refers to the role of experiential knowledge in implementation. Key: Low = low likelihood of successful implementation; High = high likelihood of successful implementation

### The ENRICH trial

A trial of peer support for discharge from inpatient to community mental health care indicated that peer support was not superior to care-as-usual (follow up by community mental health services within seven days of discharge) in terms of either the primary outcome – readmission within 12 months of discharge – or a range of secondary outcomes [26]. PWs received eight days of training focused on individual strengths and connecting to community, met the people they were supporting at least once while still inpatients and then weekly for up to four months post-discharge. Peer support was flexible and collaborative, informed by a peer support principles framework [27]. PWs received group and individual supervision from an experienced peer worker coordinator (PWC) who had access to an action learning set with other PWCs across study sites. The trial and intervention are described in detail in a protocol paper [28].

Findings from the trial indicated that 62.5% of participants offered peer support had at least two contacts with their PW, at least one of which was post-discharge, and that those participants were significantly less likely to be readmitted than a similar group of PWs in the care-as-usual group [26]. There might be many reasons why people chose not to, or were unable to engage with their PW, including the possibility that peer support was not always well implemented into practice in the trial.

## Aims

This paper aims to explore if and how levels of engagement in a new peer support intervention were associated with implementation of the intervention, and therefore how implementation of peer support in mental health services might be optimised in the future.

## Methods

### Study design

We take a comparative case study approach, informed by case-orientated Qualitative Comparative Analysis [29] and pattern-matching [30] techniques, considering the seven sites where the study took place as cases. Sites were National Health Services (NHS) mental health trusts (public healthcare provider organisations) in England, where the new peer support intervention was delivered as part of the ENRICH trial. Sites were selected to provide contrast in urban, town and rural localities, geographical spread across England, and where mental health trusts were committed to introducing new PW roles into mental health services. In most sites PWs were directly employed by the mental health trust, while in others a much smaller, voluntary (not-for-profit) sector organisation was sub-contracted by the trust to employ PWs to provide support to people using mental health trust services. Information about each site is given in Table 2 below.

**Table 2:** Characteristics of sites

	Site 1	Site 2	Site 3	Site 4	Site 5	Site 6	Site 7
Total participants/ no. offered peer support	181/93	158/78	69/34	71/33	41/21	39/21	27/11
Number (%) offered peer support with data on engagement with peer support*	89 (96%)	76 (97%)	34 (100%)	17 (52%)	20 (95%)	20 (95%)	9 (82%)
Number (%) engaged with peer support^	61 (69%)	39 (51%)	27 (79%)	8 (41%)	16 (80%)	6 (30%)	6 (67%)
Site fidelity score#	10.4	11.0	11.2	9.0	10.9	7.9	9.7
Peer worker employer	NHS	NHS	NHS	Vol	NHS	Vol	NHS
Location	Urban	Urban	Urban	Town/ rural	Urban	Urban/ rural	Town/ rural

Key: *Participants whose allocated peer worker is known and for whom data on contacts with the peer worker is therefore available; ^Participants who had at least two face-to-face meetings with their peer workers, at least one of which was in the community post-discharge; #Higher score equates to greater fidelity (range 3–12); NHS = National Health Service; Vol = voluntary (not-for-profit) sector

**Fig. 1:**
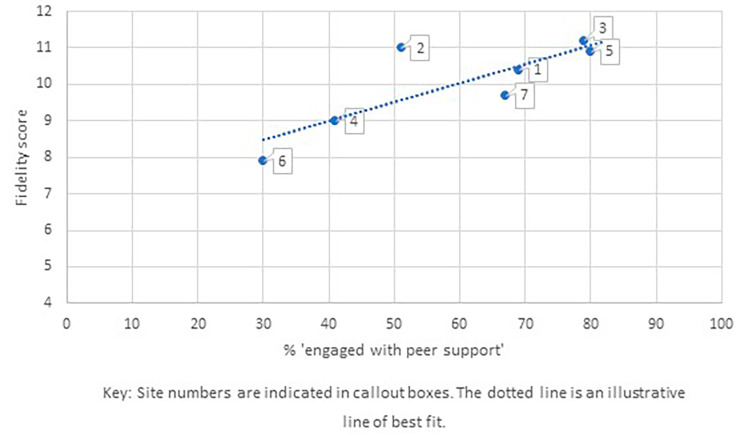
Relationship between engagement with peer support and fidelity

To inform case selection for the comparative analysis we charted level of engagement at each site – percentage of trial participants offered peer support who had at least two contacts with their PW, at least one of which was post-discharge – against site fidelity score, measured using an index designed to assess fidelity of delivery of peer support at site level against a set of principles articulating what is distinctive about peer support compared to other forms of mental health support [31] (Fig. 1). Fidelity was assessed through a semi-structured interview with PWs, the people they supported and their supervisor, rated by researchers against criteria based on the principles framework. A high fidelity score indicates that peer support had been implemented according to those principles. Fidelity was assessed after peer support had been delivered for at least six months at each site.

Figure 1 is indicative of a direct relationship between engagement with peer support and fidelity, offering rationale for selecting sites with higher or lower levels of both engagement and fidelity as cases for comparative analysis. There was one outlier, site 2, where fidelity was high (11) but engagement was mid-range (51%). We included this site in the comparative analysis as engagement might be explained by implementation issues not related to fidelity of delivery.

We report on the *Evidence* domain of the framework in a paper describing how experiential knowledge was central to developing the ENRICH peer support intervention [32]. Our research questions here are based on the *Context* and *Facilitation* domains of the framework, with context referring largely to the NHS Trust in which implementation took place (question 1), and facilitators being the PWs and PWCs who delivered the peer support (questions 2–4):

1. How did the *culture* of organisations, *leadership* (including issues of *access and authority*) and *monitoring* and *feedback* impact implementation of peer support?
2. How did PWs and PWCs feel that their *roles* were characterised?
3. How did PWs and PWCs feel they were able to exercise *flexibility* while remaining *consistent* in their approach to delivering peer support?
4. How did *experiential knowledge* underpin peer support as it was delivered at each site?

### Data sources

**Peer worker interviews.** Thirty-two PWs delivered peer support in the ENRICH trial and were invited to give written informed consent to participate in the research. All 32 consented and were interviewed after 12 months of delivering peer support. Interviews explored how well training prepared them for the role, their experiences of working as a PW, the support they received in the role and their relationship with clinical teams they worked alongside.

**Peer worker coordinator interviews.** Eight PWCs supervised PWs in the trial. Seven PWCs were themselves experienced PWs and one was a mental health nurse who shared the role with an experienced PW. All 8 PWCs gave informed consent to participate in the research and were interviewed at the same timepoint as PWs. Interviews explored PWCs’ experiences of supporting PWs, how well they thought the role was supported and organisational issues impacting delivery of peer support.

Interviews were conducted by researchers working from a perspective of having experienced mental distress and/ or having used mental health services, and played a key role in schedule development. Interview schedules can be found in the Supplementary Material file.

### Data analysis

Interviews were audio-recorded, pseudonymised at the point of collection and transcribed verbatim.

Interview data were analysed using a framework approach [33] based on the Context and Facilitation domains of the modified PARIHS framework (see Table 1). Data were first coded to the constructs within those domains, with inductive space retained to code factors not related to the framework that participants described as impacting delivery of peer support. Second, a comparative, cross-case analysis was used to look for patterns of implementation that were: A, shared across cases; B, characterised high fidelity-high engagement cases; C, characterised low fidelity-low engagement cases; D, described implementation in the outlier case [30]. Preliminary analyses were undertaken by the first author and refined through iterative rounds of discussion with the whole team.

## Results

### Characteristics of included cases

The two high engagement-high fidelity cases (sites 3,5), and the outlier high fidelity-low engagement case (site 2), were in urban areas with PWs directly employed in mental health NHS Trusts (see Table 2). The two low engagement-low fidelity cases (sites 4,6) were in areas that were a mix of rural localities with small towns or urban localities respectively. In both the latter sites PWs were employed by voluntary sector organisations outside of the NHS.

### Characteristics of participants

A total of 20 PWs were included in the analysis, five each from sites 3 and 4, three each from sites 2 and 5, and two from site 6. Twelve PWs were female, seven were male and one preferred not to say. Three PWs were aged under 35 years of age, 12 aged from 35 to 55 years, one over 55 and four preferred not to say. Eleven PWs were White British, two were White Irish, one White other, one Black/ Black British, one Asian/ Asian British, one Arab, one Mixed White Asian with two preferring not to say.

There were five PWCs, one from each site. Four PWCs were female and one was male; two were aged from 35 to 55 and three over 55; all were White British.

Participant quotes presented below are identified with a site code (e.g. S1 = site 1) and role identifier (PW = Peer Worker; PWC = Peer Worker Coordinator) plus an additional number to distinguish between PWs at each site.

A. Implementation across cases

A number of implementation features were evident across all five cases, including characterisation of the PW role as largely consistent with the principles that were used to inform development of the intervention; [27, 32] taking a non-judgemental approach and sharing experiences to create a safe space, make connections and build relationships:

‘We’re not going to be judgemental so to speak. It’s a safe place really for people to be themselves regardless of what their mental health issues are or mental health diagnoses are.’ (S5PW2).

‘I’m always sharing lived experience, whether that’s just generally or whether that’s personally with mental health … obviously you share when appropriate but you try to match that experience together so you have something in common, and then there is that mutuality and reciprocity and creating that trusting relationship.’ (S3PW3).

On the whole, training – as specified in the ENRICH manual – was consistently delivered and worked well to provide PWs with the range of skills they felt they needed for the role:

‘We did a lot about strengths-based approaches and I think that’s really informed the way that I interact with people, so I think I’m always trying to bring it back to what can you do, what is strong for you … we did a lot about active listening and also about discussing difficult issues … I think it’s been very helpful the stuff we did in training … definitely the boundaries and relationships sessions that we did …’ (S3PW1).

The importance of group supervision facilitated by the PWC, as well as individual supervision where required (both specified in the handbook), was indicated across sites, providing the opportunity for PWs to share experiences and receive feedback from one another as well as from the PWC:

‘I will hold these feelings until supervision and that’s when I let it out, offload it to my colleagues. And it’s been great because we’ve been bouncing it off each other and I’ve noticed that it’s not just me that was going through it, so it’s such a relief …’ (S2PW2).

‘… [PWC] will always ask how I am, if anything has triggered me or anything like that and she’s quite easy to talk to and it’s OK to be open with her.’ (S6PW3).

Participants in all sites described differences between the culture of clinical services in the host trust and the ethos underpinning peer support:

‘…it’s about the values because what I find with the other types of support, it all tends to be clinical and deficits based … very directive and judgemental … some of the clinical teams are stuck in that way of seeing things, that deficit-based thing and they don’t really know too much about peer support.’ (S5PW1).

At all sites, there was a perceived lack of contact with, and feedback from, community mental health teams, sometimes accompanied by a lack of understanding of the PW role:

‘Whenever I got a new service user, I’d email their [Community Psychiatric Nurse] or care coordinator … to give them more information about it and nobody, apart from I think one person, got back to me. So that’s been quite challenging, not really having any communication or contact really with the mental health teams that are working with the service users …’ (S5PW3).

Interviewees in all sites remarked that the timing of the offer of peer support - prior to discharge from hospital - was particularly challenging for some, especially in relation to maintaining contact with the PW following discharge. This represented a barrier to engagement that was related to the clinical context, rather than implementation:

‘… they are being introduced to it as soon as they come out … they are going through a tough period of fear, of not knowing what’s next for them. The last thing they want is to commit to 16 weeks of meeting someone that they don’t even know.’ (S2PW2).

‘I suspect that the post-discharge needs more targeting, that would be my sense. There are people who really get so much out of it, but then there are an awful lot who just disengage. It’s another stress for them I think.’ (S4PWC).

B. Implementation in high fidelity-high engagement cases

There was evidence of features supporting implementation in the high fidelity-engagement cases which contrasted with low fidelity-lowengagement cases (see below). In high fidelity cases, cultural differences between clinical services and peer support were generally seen as an asset and were valued, rather than as a source of tension:

‘… you need a values-based practice and how important it is, as opposed to the clinical based practice and how helpful that is … I’m not saying the clinical approach is wrong or anything like that, what I’m saying is we need to complement each other, we need to take a holistic approach.’ (S5PW1).

Some aspects of organisational culture were seen as supportive of peer support, including the role of recovery colleges in preparing PWs for the role or providing additional training once in post (recovery colleges employ an adult education model to supporting people with their mental health, often co-delivered by people using mental health services [34]):

‘… we were in a really fortunate position being linked with a Recovery College, that, where later in their work they then wanted to do specific recovery focused training around diagnosis we were able to provide that for people.’ (S3PWC).

In these cases, staff on the wards (inpatient units) were reported as largely familiar with and valuing the role of peer support:

‘… when I’d go on the ward … they seemed to see great value in the transparency of people being there because they’ve got lived experience. That aspect of it was really nice … good for the culture of the organisation in many ways.’ (S5PWC).

‘… the clinical teams are aware … they’re very excited that we’ve got peer workers on the ward. They’re very positive about it.’ (S3PW3).

PWCs described PW recruitment as having followed the process specified in the intervention handbook, and as such the PWs who were appointed were well equipped to deliver the role:

‘… we had the right people to execute these roles effectively really … we had quite a diverse selection panel … we had the right people that expressed the interest I think …’ (S5PWC).

There was evidence that PWs and PWCs – as intervention facilitators - had worked hard in delivering clinical team preparation sessions, as specified in the handbook, offering repeat sessions where necessary, and that this had supported a good relationship with ward teams:

‘… [in] the early days we went in to talk about ENRICH and then if they’d had significant staff turnover, which is really happening a lot … we’d then go back to the teams just so that they were aware of what ENRICH was about, what their role was … it certainly meant that staff were much more welcoming of the ENRICH peer workers when they came onto the wards.’ (S3PWC).

PWCs reported being well resourced in their leadership role, both in terms of having sufficient time to do the work and having sufficient supervision themselves around any difficult issues that might arise:

‘… [my role] was two days a week and that was plenty of time…’ (S3PWC).

‘I have had unconditional support from my manager … it’s been part of my regular monthly supervision … any kind of difficulties I’ve had or frustrations or whatever that has come up, that has been an ideal time to go through it. But I’ve also been supported to discuss things as and when they come up …’ (S5PWC).

In these sites, there was evidence that delivery of peer support was highly consistent with the manual. There was notable emphasis on flexible application of peer support, especially around pacing support in response to the individual’s needs, spending as much time as necessary alongside the participant to build a trusting relationship:

‘… it doesn’t necessarily follow a linear path a lot of the time. Sometimes, somebody might be having a really bad week and they actually want you to listen to what’s been going on for them … at the beginning, because you are getting to know the person as well, I think the kind of conversation you’d have is a bit more general … and then it might actually take a completely different path however many meetings down the line and they’ll actually go … “I haven’t told anyone about a particular issue, but I want to talk it through with you and see what you think”.’ (S3PW1).

PWs at these sites demonstrated confidence in taking a lead from the person they were supporting, consistent with the principles of choice and control that underpinned the intervention:

‘… I’m kind of getting to know things that they’re interested in and this is influencing where I signpost them to … it’s just about giving them the option and then they can make their own decision then whether they want to go, and again that’s putting them back in control, which is all about helping people to recover really and take control back of their lives.’ (S5PW2).

PWs also described learning from the people they were supporting, and the importance of validating their experiences, consistent with the principle of reciprocity in the underpinning framework:

‘There are people who I’m supporting who … realise that the medication is very important to them and that they will probably always be on it. So, I gain insight from that, just because maybe I found that medication in my own lived experience wasn’t particularly fantastic but for others it’s very important. So, you learn from other things … you’ve got to validate their experience because … they know what works for them and you can’t tell somebody else what will work for them …’ (S5PW1).

C. Implementation in low fidelity-low engagement cases

There was evidence of barriers to implementation in low fidelity-lowengagement cases. In both, PWs were employed in not-for-profit organisations, resulting in organisational context-related barriers to implementation. Resource issues impacted leadership of the intervention with, in one site, the organisation not having capacity to provide cover or suitable supervision for the PWC:

‘… we’ve had different staff line managing me over the past year because of maternity. But to be fair none of them really knew about ENRICH … there was nobody who could have covered my role here … it’s felt like a bit of pressure to continue doing it because I took a bit of time off … I couldn’t physically go out and do anything when I wasn’t well …’ (S6PWC).

Support for PWCs at these sites, including an Action Learning Set with other PWCs, was difficult to access because of lack of sufficient funding to travel to meetings:

‘I think the action learning sets worked really well … maybe they should have been planned for a bit more financially … because ultimately we had to go back to our Trust and say we need to find more money or I’m not going.’ (S4PWC).

Being outside of the NHS also created issues of access and authority for PWCs:

‘I would have thought there should be regular team meetings, but we never seemed to be able to get in on them … an additional disadvantage from being an organisation outside of the Trust …’ (S4PWC).

‘… it’s been difficult with the [NHS Trust], some of the staff there … I don’t want to say too much, but that’s been difficult.’ (S6PWC).

This extended to PWs being able to communicate with clinical teams about the people using:

‘A few times they didn’t want to talk to me because I didn’t have enough information for them … to establish who I was … I just wanted to know whether they were seeing [participant] or whether they’d stopped seeing him, and they wouldn’t tell me.’ (S4PW3).

At these sites there was, generally, a challenging relationship with ward-based clinical staff, potentially impacting on the initial relationship building phase of the peer support:

‘There were certainly, on that site, a lot of suspicious looks and “what on earth is this all about” type conversations. However much we tried to prepare the staff team, and we’d gone in and visited and talked to them all, but there was still that “what’s this all about”? People didn’t get it straight off.’ (S4PWC).

Cultural differences with the host NHS Trust were keenly felt by PWs employed in not-for-profit organisations:

‘… the ward environment is, well obviously it’s clinical. It sometimes feels some staff, but not all staff, who work on the wards are not really sure what my role is or have a vague understanding. There’s perhaps a little bit of a difference in terms of pecking order and me in the pecking order.’ (S6PW2).

‘… they will be looking at the patient’s files … they can build up a judgement before seeing you … when the patient sees the peer support worker they might talk to us because we’re non-judgemental, we don’t feedback unless there is a safeguarding issue or danger to themselves or others … I don’t think peers should be seeing files …’ (S4PW44).

Both sites also combined rural localities with urban areas, with issues of geography hindering timely delivery of peer support at remote hospital sites:

‘… the geography issue was a great challenge in itself in our area because I was one bit of the triangle and the [hospitals] were in two different places … I’d have had an hour or so travelling and then get there and “oh, they’re on leave until 10pm tonight”.’ (S4PWC).

Geography could also impact on building a strong sense of PW team:

‘I did lots of talking to [the PWC] but not so much my fellow peers. There was one fellow peer that I talk quite a lot to … the other two were very close to each other and so they were almost functioning as one … I got on OK with the people at [the other town] … it’s just that we had differences of opinion.’ (S4PW3).

There was some inconsistent delivery of training, with one PW reporting having received a truncated version of the training programme as a result of capacity issues:

‘I didn’t actually do [the full training] … because I was covering a maternity leave it was the girl did all the training. So, I basically had a morning with the coordinator where we went through the whole bumph together … ’ (S6PW3).

While the importance of group supervision was acknowledged in these sites, there was disruption leading to inconsistency with the pattern of weekly group supervision as a result geography in one site, and capacity in the other:

‘We don’t generally do weekly anymore … generally we do monthly although I check in by phone with them.’ (S4PWC).

‘… a lot of the supervision has ended up being one-to-one just because it’s a small team here … sometimes I would be able to meet with them together but often because my day, I’ve only got one a day week, I’d have to fit them in if one of them couldn’t do it that day …’ (S6PWC).

Possibly as a result of disruption to supervision or opportunities to support each other as a team, PWs at these sites at times appeared to lack confidence in delivering peer support:

‘… it made me feel that I was getting it all wrong … she didn’t really talk at all about, and I felt that I couldn’t, I just felt that I had to wait for her to give information to me … because that’s what I understood you are supposed to do, is wait for them to give you information to talk about their problems …’ (S4PW3).

‘… I’m imagining it’s going to be quite hard for a long time because the expression that I’ve used that comes to mind is pulling teeth. It’s going to be probably like that every time we meet … it is frustrating because you want to help them.’ (S6PW2).

D. Implementation in the outlier high fidelity-low engagement case

The outlier case shared contrasting sets of features with the other cases. Like high fidelity and engagement sites, the outlier case reported feedback from management describing a positive impact of peer support on culture in the NHS trust:

‘… within senior management they’ve seen the power of peer working and they really like it … we’re in discussions on when ENRICH finishes, that we’re going to have a number of peer workers within teams, exactly to try and change the nature and change the culture …’ (S2PWC).

PWs in this site also demonstrated a more confident, patient approach to relationship building:

‘… trying to build that friendly rapport, getting them to trust you, showing them that you understand them in a way … creating that safe space environment for them to be able to talk about how they are feeling or what’s going on for them … just finding out what they want to do for themselves not someone else telling them what to do … ’ (S2PW0).

However, as in the two low fidelity-low engagement cases, in the outlier site barriers to implementation included a challenging relationship with ward staff:

‘… [I feel] looked down upon sometimes, “oh, you’re just a peer support worker” … it’s the environment. The days that I do go for ward meetings are usually the days I need a long break, I’ll be honest with you …’ (S2PW2).

In this site there was also disruption to group supervision, with some PWs needing considerable additional support from the PWC and a challenging team dynamic emerging:

‘I was definitely doing weekly one-to-one supervisions with the peer workers when they first started … it kind of came apparent that it was what people needed … for me it didn’t work very well, I was exhausted … people want one-to-one sessions to talk about colleagues and issues they are having with their colleagues … I think there are two other peer workers who are less, they don’t see themselves as much as part of the team.’ (S2PWC).

The PWC indicated that they would have benefitted from additional support for their role:

‘I feel like we could have done more support around, more training kind of stuff on managing people with lived experience … … maybe one thing would have been more meetings with other peer worker coordinators and just see how other people are doing it … more guidance on what group supervision actually was … ’ (S2PWC).

## Discussion

This study used a qualitative, comparative case study design to explore how implementation of a peer support intervention might be associated with engagement with peer support and, as indicated in results elsewhere, [26] with outcomes. We noted clear differences related to organisational context between high fidelity-high engagement cases and low fidelity-low engagement cases. Lack of a positive working relationship between PW and ward (inpatient) clinical teams, exacerbated by lack of awareness of the potential role of peer support, is likely to be crucial to engagement where people begin peer support in hospital. Levels of engagement were highest in cases where those relationships were reported as largely positive and where differences in approach (between clinical practice and peer support) were highly valued [6, 35].

We note that the two low fidelity-low engagement sites employed PWs in the not-for-profit sector rather than within the NHS. Elsewhere, research has indicated that the principles underpinning peer support might be better maintained within peer-led or not-for-profit organisations, [7] and that doing so might provide an opportunity to bring a change of culture into statutory services [35]. However, we observed constraints on resourcing for leadership roles, and lack of access and authority for managers in the not-for-profit sector, compounded, perhaps coincidentally, by the additional challenges of geography. Neither did we observe, in those sites, evidence of leadership for peer support from within the host NHS organisation that might have facilitated better implementation [36]. In our outlier high fidelity-low engagement case, resourcing for leadership also impacted support for PWs. Proper resourcing for PWCs has been identified elsewhere as crucial to providing good peer support [5, 37]. PWCs at sites that struggled with levels of engagement identified the need for a wider network of mutual support beyond their immediate organisation, with work elsewhere highlighting the need to develop communities of practice around lived experience leadership roles in mental health services [38, 39]. As such, our findings reinforce the link that has been observed elsewhere between leadership in implementation, and the outcomes of a newly implemented intervention [40].

At the two high engagement-high fidelity sites, PWCs noted that robust recruitment processes resulted in a PW team that were well equipped to deliver what was a challenging role. An experience of the PW team as mutually supportive, complemented with group supervision led by a PWC bringing experiential knowledge to their role, was identified as important at all five sites included in our analysis, as it is in the wider literature [2, 5]. The PW training programme was equally valued across all sites with PWs indicating that it prepared them well for their roles. Again, the importance of training that is specifically tailored to peer support having been widely noted [3, 4]. In sites where there were inconsistencies in delivery of supervision and training, this appeared to impact confidence among PWs in offering peer support that reflected the underpinning principles framework. Sites with high fidelity scores were indicative of a clear focus on relationship and trust building, characterised by spending time alongside the individual offered peer support, learning from them, before taking their lead in exploring new possibilities. These values have been identified as fundamental to peer support, [41] and our own analysis of data from the trial indicated that relationship building at the beginning of the peer support was predictive of ongoing engagement [42].

It is worth noting here that not all challenges to engaging people with the peer support were attributable to implementation issues. Across sites, interviewees felt that discharge from hospital was a challenging time for some people to consider taking up peer support. Other trials of peer support for discharge have also struggled in this respect, [43] especially where participants were those with a higher level of need (people with multiple admissions) as they were in our study [15].

### Strengths and limitations

We employed a robust, theoretically informed comparative case study design, with case selection determined by a priori measures of fidelity [31] and engagement [26] made independently of this analysis. We analysed a complete data set – interviews of PWs and PWCs – in all sites included in the analysis, although we might usefully have also interviewed NHS clinicians and managers as they also played a role in implementation. Analysis of in-depth interviews exploring the experiences of people offered peer support will be reported elsewhere. Our original interview schedules were not directly informed by the PAHRIS framework [22] and so may not have elicited a full range of data relating to implementation variables. Other frameworks might have been indicative of different barriers and facilitators of successful implementation. Nevertheless, we note the work adapting the PAHRIS framework to elucidate the role of experiential knowledge in implementation was particularly suited to a study of peer support and informed by lived experience on the research team [25].

### Implications for policy, practice and research

Mental health workforce policy in England, as elsewhere, is encouraging employment of large numbers of PWs into mental health services.[44]. A range of training programmes have emerged [45] that, to some degree, share a set of principles similar to those that informed ENRICH. This study suggests that specific supports for PWs need to be properly resourced as integral to the offer of peer support in mental health services. These include supervision from an experienced PW, opportunities for group supervision, and an emphasis on relationship building in PW training that is consistent with a principles-based peer support framework. While it has been suggested that peer support can drive cultural change in mental health provider organisations, [46] our research suggests that lack of supportive culture can constrain delivery. Peer leadership, provided with sufficient support and authority, is needed to support change work with clinical teams, in hospital and in the community, so that peer support and clinical care are part of a complementary offer.

This study identifies policy and practice implications when peers are employed through not-for-profit organisations to work in partnership with public mental health providers. Research elsewhere highlights the potential challenges and opportunities of this ‘hybrid’ approach, [35, 47] indicating a need for strategies that effectively align implementation expectations between the not-for-profit organisation and the mental health provider.

Further research to develop and evaluate the introduction of peer support in mental health might usefully be informed by a change model that incorporates this range of implementation variables to optimise delivery of peer support. We also note that in our study, PWs were employed to, and supervised within a dedicated PW team that provided peer support across several clinical teams, while in many mental health services internationally PWs are employed as embedded members of multi-disciplinary clinical teams. There is a need for research that considers the implications for implementation and outcome of these contrasting organisational configurations.

## Conclusions

This study provides clear indication that implementation issues can impact delivery of peer support, with implications for engagement and, potentially, outcomes. Resourcing can impact consistency of delivery, alongside challenges of access, authority and relationship with clinical teams, especially where PWs are employed outside of the mental health service. Attention needs to be paid to the impact of geography on implementation.

## Electronic supplementary material

Below is the link to the electronic supplementary material.

Supplementary Material 1

## Data Availability

The datasets used and analysed during the current study are available from the corresponding author on reasonable request.
