# Supplementary material for: Implementing peer support into practice in mental health services: a qualitative comparative case study
Source: BMC Health Serv Res. 2024 Sep 11;24:1050. doi: 10.1186/s12913-024-11447-5 (PMC11391751; doi:10.1186/s12913-024-11447-5)
Supplement: Supplementary file 1 — Supplementary Material 1 [file 12913_2024_11447_MOESM1_ESM.docx]

**PEER WORKER INTERVIEW SCHEDULE**

**12 MONTHS**

# Can you describe a typical conversation you might have with a peer during your first few meetings? Reflecting on the last year, what works best about those initial conversations in terms of supporting people with discharge?

- - What sort of things do you often talk about?

-What sort of things might you share with each other?

- - How do these conversations feel for you?
  - How much is the focus on the experience of being discharged and how much is about building relationships?

# Thinking about typical sessions you have with a peer when you are working together in the community, can you describe what sort of conversations do these sessions often involve?

- - Does the nature of those conversations change over the time you work together, and if so, how?
  - Can you give some examples of things you’ve done with peers?

# What kinds of activities do you do with your peers and where do you tend to meet?

- - Who chooses / finds them (you / supported peer)? What tends to be the focus of the activities you do? Who runs them (i.e. voluntary / statutory)?

# Thinking about sharing lived experience in your role, how often do you find that you share your personal experiences with the peers you work with? Has that changed over time?

- - What sort of things do you share with them? Is this always reciprocal?
  - How do you decide what to share, or how much to share with a peer?
  - Can you think of any examples where sharing went particularly well? Or any examples where this has been difficult for any reason?
  - Do you think that sharing your personal experience with a peer makes a difference to forming a relationship with them? If so, in what ways?
  - Do you think that sharing has any impact on the peers you work with, and if so, what sort of impacts?

# Thinking about some of the peers you have worked with, what has it been like to explore their experiences and understandings of mental health?

- - Have there been any examples where you have learned something new from your peer’s

perspective?

- - Has a peer’s personal understanding of mental health ever challenged your own

understandings in any way? What impact do you feel this may have on the relationship?

1. **Have there been times when peers haven’t taken up the offer of peer support? (If so,) how has**

**that felt? What ideas do you have about why this happens?**

1. **What things help you to connect with peers?**
   - Have there been times when it has been more challenging to build up a relationship with peers? Has anything helped you to cope at these times? (Personally and in your work with your peer.)

# How have you found ending the peer support relationship with people?

- - What can help this go well?
  - Have there been any challenges?
  - Are there additional things you would have found useful in the training which might have helped with this?

# Do you feel the peer support relationship has had an impact on the way your peers choose to relate to others?

- - For instance, the way they want to relate to family, friends and wider community? Can you give some examples?
  - Or the way your peers might relate to professionals in clinical teams? Have you been part of observing or hearing about that at all? Do you feel the peer support relationship has had an impact on this?
  - How much of the time do you focus on building connections/relationships with people?
  - Has it also been important to spend time identifying relationships that are unhelpful or negative for your peer? And if so, what to do in this situation?
  - Do you spend any time working with your peers around feelings of stigma and

discrimination they’ve encountered from others?

# Have you noticed any changes in the ways peers talk about themselves (how they ‘see themselves’?

- - Do you think peers reflect on their own strengths and values during your work together?
  - How about self-esteem and self-confidence?
  - How about feelings of hope for the future?

-Have you ever noticed any barriers that often stop your peers from connecting to people and/or places in their communities? What sort of things have you noticed? Are there any ways around this that you have discovered?

-Have you noticed changes in the ways peers make decisions over time?

**Do you think *the peer support relationship* has played a part in how peers talk about themselves**

# (how they ‘see themselves’)?

- What is it about what you do together that you think might have contributed towards this?

1. **Do you think the peer support offered is different to other types of support? If so, how? Do you think your peers would agree?** (Support from family/friends etc but also professional support)
   - Do peers tell you about any changes in how they’re choosing to use various services (NHS, voluntary etc) / or the way they are using services (frequency / speaking up about what they want, etc.)?

# How do you think your peers view you? Do you think being a paid member of staff has an impact on the peer support relationship? (If so,) how?

1. **Overall, what do you think the key aspects of successful peer support are?**
   - What makes you feel that aspect is particularly important….?

# How have you found the Peer Worker role in the last few months now you have been in the role for about a year?

- - How has the role changed in that time?
  - Have there been changes in the peer worker team? Has staff turnover or sickness had an impact on your role?

# What do you enjoy about this role?

1. **What are the challenges of doing this role?**
   - Has the role met your expectations?
   - Has anything surprised you about the job that you weren’t expecting?
   - Has there been anything positive aspect of the job that you weren’t expecting?

Has there been anything disappointing about the job that you weren’t expecting?

# How much contact/interaction do you have with Trust clinical teams (such as CMHTs, Care Coordinators etc)?

-How do those interactions affect the job you do?

-What works well about those communications/interactions?

-What could be different?

- How have you found working within NHS hospitals? Have your attitudes or opinions changed as a result?

# Since doing this role, have your perceptions of the mental health system changed?

- - If you are using services currently, has your work affected the way you use services?

# How helpful is the support and supervision you receive from the Peer Worker Coordinator in doing your role?

- - What works well about support and supervision?
  - What might be different or is there anything you would change?

# How helpful is the support you give each other as a Peer Worker team?

- - What works well about that team support?

What could be different?

# Have there been any situations where you felt worried about one of your peers or particularly difficult things which have happened? (E.g. ‘risky’ situations)

- - What impact has that had on you?
  - What support has helped with this? Is there anything else you would have wanted to be put in place?

# In what ways do you look after yourself so that you stay well in this job?

- - Have you been confident in taking sick leave when you have needed to for managing your wellbeing?
  - Have you been well supported during times of sickness? How were you supported in your return to work?

# Are there any additional things that could help your well-being at work?

- - In what ways has working as a peer worker affected other aspects of your life?

1. **Has this role made you think about future working opportunities or had an impact on your future plans in any way?**

**PEER WORKER COORDINATOR INTERVIEW SCHEDULE**

**12 MONTHS**

1. **How well do you think Peer Workers have been able to adjust to and carry out their role?**
   - What is going well, and why?
   - What isn’t going so well (and why)?
   - What might have happened differently, and how? (e.g. activities, skills, resources for peer workers)
2. **Looking back to training now, how well do you think it prepared peer workers for the role?**
   - Are there things it would have been helpful for peer workers to cover at the time or additional training at a later time which would have helped Peer workers in their role?
3. **How much (extra) support and supervision was provided to the peer workers during their first weeks in the new job? Is there flexibility around the support that can be given?**
4. **How much 1-1 supervision have you provided and how has that worked?**
   - Are there particular times when peer workers have requested / benefited from 1-1 support?
   - Has there been enough time / flexibility for you to be able to provide the supervision peer workers need?
5. **How has the sense of team been among peer workers?**
   - How useful do you think group supervision has been in supporting peer workers to do their jobs?
6. **What demands and challenges do you feel that Peer Workers have encountered in their role?**
   - In what ways have they responded to these demands and challenges?
   - Is there anything which you think might have helped with this?
7. **In what ways have Peer Workers been successful in the role?** (Please give some examples.)
8. **Overall across the team, have there been any aspects of the role that might have**

**impacted negatively on the team’s mental health?**

- - (If so,) what support has been helpful?
  - Could anything else have been put in place which may have helped (e.g. changes to the scope / demands of the peer worker role, extra supervision, other sources of support / training?)

1. **How much contact do the peer workers have with clinical teams (inpatient and community)?**
   - In what ways have these interactions impacted on the peer support given?
   - What might have been done differently in preparing the clinical teams and peer workers to work together?
   - Have clinical teams supported you to undertake your role in leading the peer worker team? (If so, what have they done / said which has helped? How much do you feel they understand your role and the peer workers’ role?)
2. **To what extent, if any, do you think the Peer Worker role is having a positive impact in terms of changing the culture within inpatient and community mental health services?**
   - Are there further opportunities to support the development of that culture?
3. **How has your role been?**
   - Have there been particular challenges?
   - What things have gone particularly well?
4. **Can you describe your line management and supervision arrangements for the Peer Worker Coordinator role?**
   - To what extent have you been supported to use your lived experience in the role?
   - (And/ or) how might you have been supported differently to use your lived experience in the role?

Is there anything else that you would have liked to have in place which would have helped you to do your role?

1. **Are there things you have learnt through your involvement in this project which you will take forward and use in your future work?** (e.g. reflective learning, models of peer support)
2. **Beyond the end of the ENRICH study, are there plans for your role / the peer workers roles?**
   - Has anything surprised you about the way the study has worked in practice?
   - Is there any particular learning which has come from the study? (Suggestions for improvements / changes / organisational structures and support, etc.)
3. **Is there anything else you would like to tell us about how peer workers have found their work and the impact the role has had for them?**
